# Mortality and morbidity predictors in older persons with mild traumatic brain injury: A district-wide study

**DOI:** 10.1007/s10143-025-04014-x

**Published:** 2026-01-05

**Authors:** Lotem Kehati, Amit Azriel, Mai Ofri, Chaya Bracha Gordon, Elad Avraham, Amit Frenkel, Yuval Sufaro

**Affiliations:** 1 Department of Neurosurgery, Soroka University Medical Center, Yitzhack I. Rager Blvd 151, Be’er Sheva, 8410501 Israel; 2 General Intensive Care Unit, Soroka University Medical Center, Yitzhack I. Rager Blvd 151, Be’er Sheva, 8410501 Israel; 3 Clinical Research Center, Soroka University Medical Center, Yitzhack I. Rager Blvd 151, Be’er Sheva, 8410501 Israel; 4 Faculty of Health Sciences, Faculty of Health Sciences, Ben-Gurion University of the Negev: Caroline House, Ben-Gurion University of the Negev Be’er Sheva, Be’er Sheva, 8410501 Israel

**Keywords:** Traumatic brain injury, Older adults, Mortality, Outcomes, Anticoagulants

## Abstract

As the global population of older adults grows, mild traumatic brain injuries (mTBIs) pose an increasing health concern in this demographic. We aim to identify factors influencing mTBI outcomes and to better understand the mortality rates in older patients with mTBIs. We conducted a single-center retrospective analysis of 7,218 patients aged 65 + with mTBI at Soroka University Medical Center (2000–2020). Our study employed a rigorous definition of mTBI (GCS 14–15 only), reflecting institutional protocols and enabling focus on the mildest TBI spectrum. Univariate tests were utilized to compare patients with all-cause mortality within one year following the mTBI and patients who survived beyond the first year of injury. The Kaplan Meir method was used to assess one year survival along with the log-rank test to evaluate survival differences. A COX proportional hazard regression was performed as well to identify potential risk factors for 1- year all-cause mortality. One-year overall mortality rate was 18.7%. Multivariate analysis identified several significant independent predictors of increased mortality, including prior TBI, pathological neuroradiological findings, two or more comorbidities, and chronic use of Novel Oral Anticoagulants (NOACs). In contrast, chronic use of Warfarin and antiplatelet agents was not associated with increased mortality risk. This study sheds light on the complex interplay between clinical factors and outcomes in the mildest spectrum of TBI in older adults. It emphasizes the importance of tailoring treatment plans based on individual risk factors to enhance clinical decision making. Using refined risk stratification criteria has the potential to improve outcomes for older patients with mTBI.

**Supplementary Information:**

The online version contains supplementary material available at 10.1007/s10143-025-04014-x.

## Introduction

Mild traumatic brain injuries (mTBIs) are a prevalent subset of traumatic brain injuries (TBIs) and are clinically classified using the Glasgow Coma Scale. mTBIs significantly impact adults due to the increasing incidence of fall-related TBIs in this demographic and the poorer outcomes observed in older patients with TBIs [5, 10, 19, 25]. Older patients can be more susceptible to injury after a TBI due to increased vulnerability of their vasculature and white matter to injury and the presence of pre-existing neurological or systemic comorbidities [19].

Rapidly progressing medical and pharmacological developments are setting the stage for a significant increase in the older population with projections that by 2050 the older population in the US will double [19]. One out of three individuals over sixty-five experience a fall each year, and the high number of older adults driving increases their risk of involvement in motor vehicle accidents [5, 19, 25]. It is also notable that polypharmacy in older patients, a common occurrence, is a known risk factor for falls, morbidity, and mortality in older adults which contributes to the risk of TBIs in older adults [7, 10, 12]. Considering this, it is important to gain a better understanding of medical issues common in the older population, such as mTBI, to improve prevention and management strategies.

Despite the growing issue of mTBI in older patients, there are few evidence-based geriatric TBI guidelines available for acute or long-term management [10]. Furthermore, there is limited data regarding mTBI, the most common type of brain injury in all age groups [1, 2, 7–9, 11–15, 17, 20–24, 27, 28].

Our goal is to analyze both clinical and demographic factors that may impact mortality and morbidity rates following mTBI in older adults.

## Materials and methods

### Study population and data collection

We conducted a retrospective cohort study at Soroka University Medical Center (SUMC), using linked electronic health records from SUMC and Clalit Health Services (CHS). SUMC is a 1,191-bed tertiary medical center, part of the CHS hospital network, and the only tertiary hospital in southern Israel, delivering comprehensive acute and follow-up care. CHS is the largest integrated payer-provider medical organization in Israel, insuring approximately 5,000,000 individuals and providing a full spectrum of medical services.

In our study, we included patients aged 65 and older who visited the emergency department (ED) at SUMC between the years 2000–2020 and received a diagnosis of head trauma (Supplementary Table 1) with a GCS of 14–15 or were discharged from the ED. We excluded patients with mTBI were not members of Clalit Health Services (CHS), preventing reliable ascertainment of pre-injury history and long-term vital status. We also excluded admitted patients lacking documented GCS scores, based on our institutional protocol where GCS ≤ 13 necessitates admission. In cases where a patient sustained multiple head injuries within one month, we included the first injury. If the patient died within one year of an injury or injuries, we included the first injury within the year preceding the death.

## Study objectives

The primary objective was to identify clinical and demographic risk factors that increase 1-year mortality rates in older patients with mTBI. The primary outcome was all-cause mortality within the first year following mTBI.

The secondary objective was to investigate the association between commonly used antithrombotic medications and 1-year mortality in older adults with mTBI.

### Statistical analysis

Demographic, clinical and pharmacological variables are presented as mean ± SD for normally distributed variables, median and inter quartile range (IQR) for non-normally distributed variables, and absolute counts with percentages for categorical variables. Univariate tests were performed to compare patients with all-cause mortality within one year following the mTBI and patients surviving beyond the first year after their injury. The Student’s T-test or Mann-Whitney was used for normally distributed parameters or for non-normally distributed variables, respectively. Chi-square test or Fischer-exact test was used for categorical variables.

One year survival analysis was assessed using the Kaplan-Meier method. The log-rank test was used to evaluate the differences in survival. COX proportional hazard regression was performed to estimate potential risk factors for 1-year all-cause mortality, accounting for variables with clinical or statistical significance (*p* < 0.1). The assumption of proportional hazards was verified for all covariates in the final model using graphical methods (inspection of: log(-log(Survival)) plots). A hazard ratio with a 95% confidence interval (CI) was applied. To control multiple comparisons across the 40 statistical tests, the Bonferroni correction was applied by setting the family-wise error rate at an adjusted significance threshold of alpha = 0.00125 (0.05/40). Statistical analysis was performed using R version 4.2.0.

The ethics committee of Soroka University Medical Center approved the study. Informed consent was waived given the retrospective nature of this study.

## Results

Our study consisted of 7218 patients, all aged 65 years or over, with the diagnosis of mTBI after being admitted to the ED. The mean age of the cohort was 79.12 ± 7.85 years. Majority of the patients were female, making up 59.4% of the patients.

### Baseline characteristics

Most patients had two or more comorbidities (70.3%). The most prevalent chronic illnesses were hypertension (*n* = 5108; 70.8%) and dyslipidemia (*n* = 4593; 63.6%). Chronic antithrombotic therapy in this cohort consisted of antiplatelet medications (*n* = 2238; 31%), anticoagulation medications (*n* = 880; 12.2%) and novel oral anticoagulants (NOACs) (*n* = 442; 6.1%).

11.7% (*n* = 846) of patients admitted to the ED were found to have pathological head CT on evaluation, defined as presence of any intracranial hematoma or any type of skull fracture (Supplementary Table 1). 67.5% (*n* = 4869) were discharged from the ED without hospitalization and 18.7% (1356) died within 1 year of their mTBI, with a median time of 116 days between injury and death. Additional baseline characteristics of this cohort are presented in Table 1.

**Table 1:** Baseline characteristics of elderly patients with mTBI admitted SUMC ED between 2000–2020 and univariate analysis of mortality within 1 year following mTBI

	Overall	No death in first year	All-cause one year mortality	*p*
n	7218	5862	1356	
Gender - Male (%)	2929 (40.6)	2247 (38.3)	682 (50.3)	< 0.001
Age (mean (SD))	79.12 (7.85)	78.16 (7.52)	83.28 (7.90)	< 0.001
Comorbidities				
Diabetes Type 2 (%)	2917 (40.4)	2306 (39.3)	611 (45.1)	< 0.001
Ischemic Heart Disease (%)	2508 (34.7)	1887 (32.2)	621 (45.8)	< 0.001
Hypertension (%)	5108 (70.8)	4031 (68.8)	1077 (79.4)	< 0.001
Renal Failure (%)	759 (10.5)	505 (8.6)	254 (18.7)	< 0.001
Dyslipidemia (%)	4593 (63.6)	3691 (63.0)	902 (66.5)	0.015
Heart Failure (%)	1043 (14.4)	689 (11.8)	354 (26.1)	< 0.001
Atrial Fibrillation (%)	1309 (18.1)	936 (16.0)	373 (27.5)	< 0.001
2 comorbidities or more (%)	5071 (70.3)	3995 (68.2)	1076 (79.4)	< 0.001
Pathological brain CT on admission (%)	846 (11.7)	596 (10.2)	250 (18.4)	< 0.001
ED outcome (%)				< 0.001
Admission	2225 (30.8)	1655 (28.2)	570 (42.0)	
Discharged	4869 (67.5)	4126 (70.4)	743 (54.8)	
ICU	124 (1.7)	81 (1.4)	43 (3.2)	
Hospitalization days (median [IQR])	5.00 [3.00, 9.00]	5.00 [3.00, 8.25]	5.00 [3.00, 11.00]	< 0.001
Discharge destination (%)				0.952
Home	797 (81.1)	553 (80.8)	244 (81.6)	
Another department	150 (15.3)	106 (15.5)	44 (14.7)	
Other hospital	36 (3.7)	25 (3.7)	11 (3.7)	
4 or more visits to ED in the following year after TBI (%)	412 (5.7)	404 (6.9)	8 (0.6)	< 0.001
Days from TBI to death (median [IQR])	954.00 [258.00, 2057.00]	1568.00 [909.00, 2578.00]	116.00 [35.00, 238.00]	< 0.001

### Clinical outcomes

#### Predictors of 1-year mortality

**Fig. 1:**
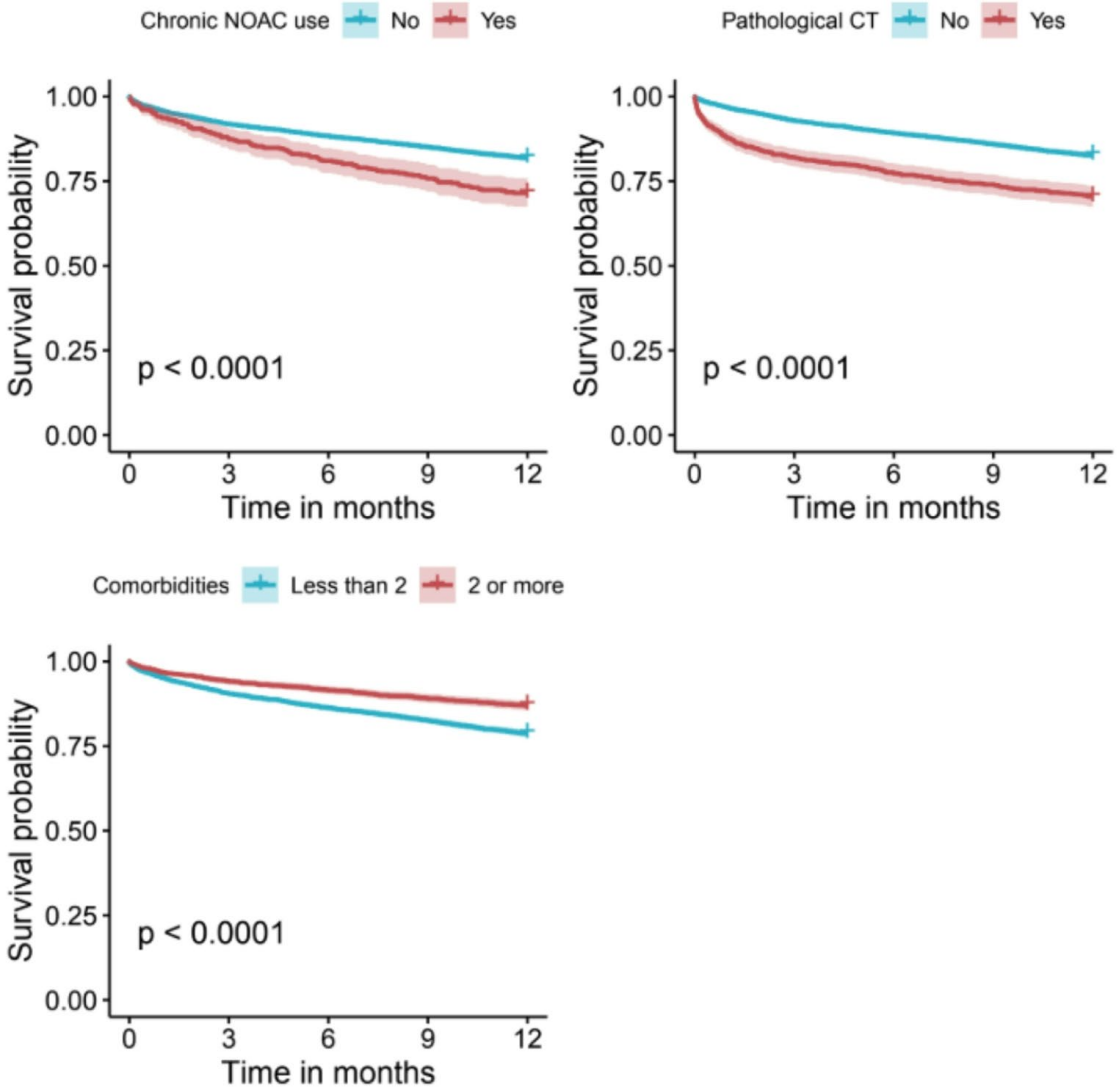
Kaplan meir curve showing the impact of NOAC use, pathological CT results, and comorbidities on survival probability after mTBI

In the univariate analyses, older age, male gender, and investigated comorbidities were all associated with increased rates of 1-year mortality in the year after mTBI (Table 1; Fig. 1). Several pathological findings on admission, including neuroradiological findings (*p* < 0.001), lower levels of hemoglobin (*p* < 0.001), pathological coagulation studies (PT-INR, aPTT) (*p* < 0.001) and elevated levels of creatinine (*p* < 0.001) were also found to be associated with an increased risk of 1-year mortality following mTBI (Table 2; Fig. 1).

**Table 2:** Univariate analysis of laboratory findings on admission of mortality within 1 year following mTBI

	Overall	No death in first year	All-cause one year mortality	*p*
n	7218	5862	1356	
White Blood Cells (#/µL) (median [IQR])	8.82 [6.96, 11.36]	8.80 [7.00, 11.18]	8.91 [6.77, 12.11]	0.553
Hemoglobin (g/dL) (mean (SD))	12.45 (1.91)	12.69 (1.74)	11.77 (2.17)	< 0.001
Platelets (#/µL) (median [IQR])	221.00 [175.00, 274.00]	221.00 [179.00, 273.00]	217.00 [165.00, 276.00]	0.096
PT INR (s) (median [IQR])	1.07 [0.99, 1.21]	1.05 [0.98, 1.18]	1.11 [1.02, 1.29]	< 0.001
aPTT (s) (median [IQR])	28.10 [24.50, 31.60]	27.80 [24.00, 31.30]	28.75 [25.58, 32.60]	< 0.001
Fibrinogen (mg/dL) (median [IQR])	473.00 [401.75, 557.00]	469.00 [403.00, 548.00]	484.00 [400.00, 578.00]	0.071
Glucose (mg/dL) (median [IQR])	126.00 [106.00, 163.00]	125.00 [106.00, 160.00]	129.00 [106.00, 171.50]	0.046
Creatinine (mg/dL) (median [IQR])	0.93 [0.74, 1.24]	0.90 [0.72, 1.17]	1.06 [0.80, 1.52]	< 0.001
Sodium (mmol/L) (mean (SD))	138.00 [135.00, 140.00]	138.00 [136.00, 140.00]	138.00 [135.00, 140.00]	0.190
Potassium (mEq/L) (mean (SD))	4.30 [4.00, 4.70]	4.30 [4.00, 4.60]	4.40 [4.00, 4.80]	< 0.001
Pulse (BPM) (median [IQR])	78.00 [68.00, 89.00]	78.00 [69.00, 89.00]	79.00 [67.00, 91.00]	0.406
Oxygen saturation (%) (median [IQR])	97.00 [95.00, 99.00]	97.00 [95.00, 99.00]	96.00 [94.00, 98.00]	< 0.001
Systolic blood pressure (mmHg) (mean (SD))	148.20 (26.98)	149.99 (26.02)	141.31 (29.41)	< 0.001
Body temperature (Celsius) (median [IQR])	36.70 [36.50, 36.90]	36.70 [36.50, 36.90]	36.70 [36.50, 36.90]	0.178

**Fig. 2:**
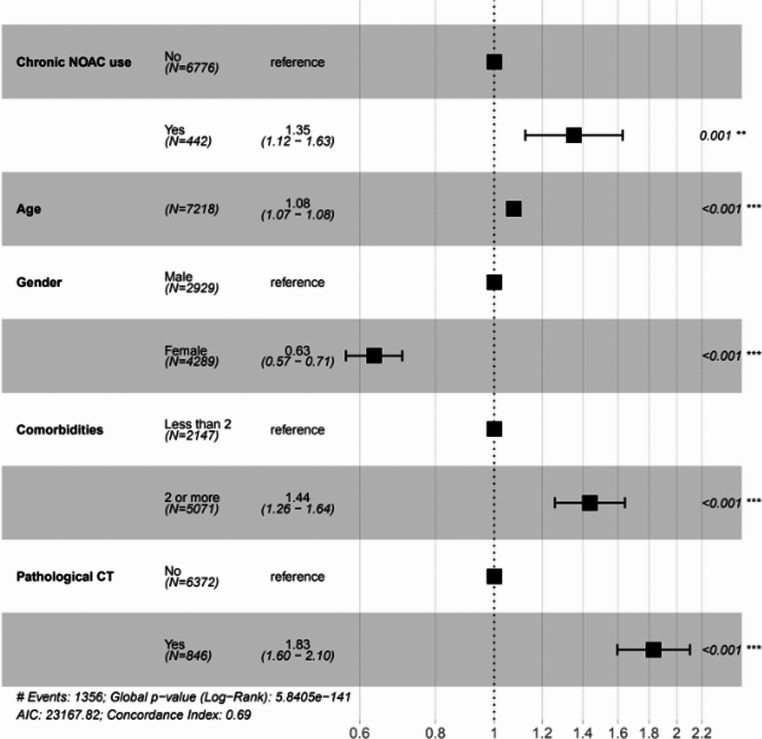
Multivariable cox proportional hazards analysis of 1-year mortality following mTBI in older patients

In the multivariate analysis (Figs. 1 and 2) we found the most significant predictors for 1-year mortality following mTBI were pathological CT findings on admission (HR 1.83; 95% CI 1.60–2.10) and having 2 comorbidities or more (HR 1.44; 95% CI 1.26–1.64). Female sex decreased the risk for 1-year mortality following mTBI (HR 0.63; 95% CI 0.57–0.71) and older age was found to be associated with increased mortality in the first year following mTBI (HR 1.08; 95% CI 1.07–1.08).

#### Antithrombotic medications (Table 3)

**Table 3:** Univariate analysis of antithrombotic medications of mortality within 1 year following mTBI

	Overall	No death in first year	All-cause one year mortality	*p*
n	7218	5862	1356	
Antiplatelets chronic treatment (%)	2238 (31.0)	1807 (30.8)	431 (31.8)	0.512
Aspirin (%)	2075 (28.7)	1678 (28.6)	397 (29.3)	0.656
Clopidogrel (%)	238 (3.3)	185 (3.2)	53 (3.9)	0.189
Dual antiplatelet treatment [DAPT] (%)				
Aspirin & Clopidogrel (%)	87 (0.9)	75 (1.0)	12 (0.8)	0.689
Anticoagulants chronic treatment (%)	880 (12.2)	647 (11.0)	233 (17.2)	< 0.001
Warfarin (%)	474 (6.6)	371 (6.3)	103 (7.6)	0.102
Enoxaparin (%)	42 (0.6)	25 (0.4)	17 (1.3)	0.001
NOACs chronic treatment (%)	442 (6.1)	316 (5.4)	126 (9.3)	< 0.001
Dabigatran (%)	136 (1.9)	101 (1.7)	35 (2.6)	0.047
Apixaban (%)	321 (4.4)	225 (3.8)	96 (7.1)	< 0.001

We found that chronic usage of antithrombotic medications was associated with increased rates of 1-year mortality following mTBI (*p* < 0.001). It is of note that the univariate analysis exhibited variability between the different antithrombotic medications. Within the anticoagulant subgroup, warfarin was not associated with an increased risk for 1-year mortality (*p* = 0.102) while Enoxaparin was (*p* = 0.001). Within the antiplatelets subgroup, none of the medications were associated with increased risk for mortality in the first year. Furthermore, chronic Dual Antiplatelet Therapy (DAPT) was also examined and did not demonstrate a statistically significant association with 1-year mortality (*p* = 0.689). Chronic treatment with NOACs, however, was found to be associated with higher rates of 1-year mortality (*p* < 0.001). In the multivariate analysis (Fig. 2) we observed chronic use of NOACs as a single antithrombotic agent increased the risk of 1-year mortality following mTBI (HR 1.35; 95% CI 1.12–1.63) (Fig. 1).

## Discussion

Given the increasing population of older adults worldwide and the significant morbidity and mortality associated with mTBIs in this age group, this study provides valuable insights into how various factors influence clinical outcomes [3, 4, 7, 10, 18]. Our study employed a rigorous definition of mTBI (GCS 14–15), excluding GCS 13 patients to specifically focus on the mildest end of the TBI spectrum, reflecting our institutional protocol where GCS ≤ 13 necessitates mandatory admission due to higher clinical suspicion.

The main objective of our study was to investigate the influence of several clinical and demographic factors on outcomes of older adults with mTBI, as previous studies results lacked focus on mild TBI specifically in older adults [2, 11–15, 21, 23, 24, 27]. We found that comorbidities, pathological neuroradiological findings and abnormal laboratory findings on admission were all associated with unfavorable outcomes. Some of these findings correspond with those of previous studies [12, 14]. We found that the most significant independent predictors identified for 1-year mortality were pathological neuroradiological findings on admission (HR 1.83) and the presence of two or more baseline comorbidities (HR 1.44). This translates clinically to an 83% increase in the hazard of death over the year following mTBI for patients presenting with pathological acute imaging, and a 44% increase for those with significant chronic health burden. These findings underscore the critical prognostic value of both acute injury severity and baseline health status in this vulnerable population, giving the fact that age-related comorbidities also contributed to the greater burden of TBI hospitalization among older adults.

Increased age was identified as a relative risk factor for 1-year mortality, with an 8% increase in mortality risk for every additional year of age. This finding correlates with those of Seno et al. [23], who demonstrate that in patients with mTBI 75 years of age and older, age is not associated with poorer outcomes. Steyerberg et al. [24] and Bobeff et al. [3] had similar correlations between age and poorer outcomes in TBI patients but lacked focus on mild TBI specifically in older adults.

In addition to examining the effects of several factors on 1-year mortality, we also investigated the effect of chronic antithrombotic and anticoagulation use on mTBI outcomes. These medications are frequently used by older adults for their increased risk of thromboembolism, but in the context of falls and TBI it may cause a serious dilemma for clinicians who must balance that risk with the risk of intracranial bleeding and other complications. Although antithrombotic agents have been investigated in the context of TBI outcomes, previous studies have yielded conflicting results [6, 8, 13, 16, 17, 22, 26, 28]. Importantly, while the inherent limitations of our retrospective administrative data preclude reliable ascertainment of specific NOAC indications, the observed correlation remains a crucial descriptive alert regarding this exceptionally high-risk subgroup.

We observed a significant association between chronic antithrombotic use and increased mortality risk. However, this association varied substantially across medication classes. Neither single antiplatelet agents, nor combination therapies such as Dual Antiplatelet Therapy (DAPT) or Warfarin demonstrated a statistically significant increase in mortality risk. Conversely, chronic use of NOACs was significantly associated with elevated mortality risk. Multivariate analysis confirmed that chronic NOAC use (mainly Apixaban but also Dabigatran) as a single antithrombotic agent independently increases the risk of one-year mortality following mTBI (HR 1.35). This indicates that patients on chronic NOAC therapy face a 35% higher hazard of death in the year following a mild head injury, highlighting this easily identifiable group as requiring increased clinical vigilance. It is noteworthy that the association observed with chronic Enoxaparin use is strictly considered exploratory and hypothesis-generating, and must be interpreted with caution, necessitating confirmation in larger, well-powered cohorts.

Santing et al. [20] found that older patients with mTBI patients on chronic NOACs therapy had a significantly lower risk of traumatic intra-cerebral hemorrhage (tICH) and neurosurgical interventions compared to those on Vitamin K antagonists (VKAs), and these findings appear to contradict ours. It is possible that the discrepancy arises from variations in studies objectives and definitions. First, Santing et al. pooled all NOACs in one group whereas we investigated each NOAC medication (Dabigatran and Apixaban) separately. Secondly, their main investigated outcome was presence of tICH and not mortality within 1 year but their results of in-hospital mortality following mTBI found no differences between NOACs and VKA users. Batey et al. [1] found that in-hospital mortality rates in older patients were similar across the different anticoagulants groups and that NOACs do not worsen ICH and hospital duration of stay. Müller et el [15]. showed that pre-injury NOACs use is safer than VKA in a matter of bleeding complications but had shown no statistically significant differences between these two groups concerning in-hospital mortality and ICU admission. Scotti el al. [22] found that risk of mortality was not associated with single antiplatelet use but was notably high with 2 anti-platelets, warfarin and NOACs use. It is noteworthy that those studies were based on small cohorts of patients and were not designed to focus on mTBI. In their study, Van Den Brand et al. suggested that pre-injury anti-platelets use is associated with an increased incidence of tICH [26], which seems to contradict our findings, but this metanalysis only demonstrated an association between pre-injury anti-platelets use and increased incidence of tICH with no consideration of mortality and morbidity. Other metanalyses demonstrated no significant difference between preinjury use of NOACs versus VKA for in-hospital mortality, surgery rates, and incidence of ICH progression [6, 16], findings that may correspond better with ours.

Our study was designed to include a large and diverse cohort of participants, which allowed for greater robustness and generalizability of findings, thus enabling more accurate and dependable interpretations.

There are some limitations to our study. Given the retrospective nature of the study, there may be recall or selection bias present. Additionally, there may be confounding factors that were not accounted for in the study and may have influenced the observed associations. Also, as our administrative databases do not provide granular, validated cause-of-death data, we were unable to analyze cause-specific mortality. Consequently, the survival analysis might overestimate mortality risk, as competing risk analysis requires knowledge of the specific cause or event type for all deaths. Finally, the one-year follow-up period might not capture the long-term effects of mTBI on older adults. Further work can be done to explore the potential mechanisms for the observed risk factors for mortality and morbidity following mTBI in the older population.

The association between NOACs and increased mortality warrants further investigation to understand the underlying mechanisms and potential risks in this specific patient population. Future prospective studies may establish causal relationships between the identified factors and outcomes and investigate the long-term effects of mTBI on older patients.

## Conclusion

This study provides valuable insights into the interplay of clinical variables influencing outcomes in a large cohort of older adults with mTBI. We identified several factors that were significantly associated with increased one-year mortality, including pre-injury use of specific NOACs, significant comorbidities, abnormal laboratory values, and pathological findings on head CT. In contrast to previous studies, we found that chronic use of warfarin is not associated with poorer outcomes. By delineating key risk factors in older patients with mTBI, this study may inform clinical decision-making by providing insight into the presentation of patients at increased risk for mortality or morbidity.

## Supplementary Information

Below is the link to the electronic supplementary material.

Supplementary Material 1 (DOCX 14.4 KB)

## Data Availability

No datasets were generated or analysed during the current study.

## Declarations

Previous Presentations: The preliminary results of this study were presented at the Annual meeting of the Israel Neurosurgical Society (2023) as an oral presentation.

This study was conducted as part of the requirements for MD degree from the Goldman Medical School at the Faculty of Health Sciences, Ben-Gurion University of the Negev.
