# Supplementary material for: Mortality and morbidity predictors in older persons with mild traumatic brain injury: A district-wide study
Source: Neurosurg Rev. 2026 Jan 5;49(1):102. doi: 10.1007/s10143-025-04014-x (PMC12769698; doi:10.1007/s10143-025-04014-x)
Supplement: Supplementary file 1 — Supplementary Material 1 (DOCX 14.4 KB) [file 10143_2025_4014_MOESM1_ESM.docx]

**Supplementary Table 1**

| **Diagnoses** | **ICD-9 codes** |
| --- | --- |
| Traumatic brain injury | 95909, 95901, 920.xx, 850.xx, 851.xx, 854.xx |
| Intracranial hemorrhage (intracerebral, subrachnoid, subdural) | 430-432, 4321 |
| Extradural hemorrhage | 8524, 8525, 85252, 85254, 85255, 85259, 85256, 85241, 85253, 85240, 85251, 852 |
| Skull fractures | 8003, 8004, 8044, 8044, 8043, 80411-80416, 80419, 80424-80426, 80429, 80431-80436, 80439, 80030-80036, 80039, 80040-80046, 80049, 8005, 80050-80056, 80059, 8006, 80060, 80061, 8037, 8047 |
| base of skull fractures | 801, 8010, 80100-80106, 80109, 80110-10116, 80119, 80124, 8012, 80120-80126, 80129, 8011, 8013, 80130 |
